# Resilience and Self-Care in Patients with Inflammatory Bowel Disease: A Multicentre Cross-Sectional Study in Outpatient Settings

**DOI:** 10.3390/jcm14113868

**Published:** 2025-05-30

**Authors:** Daniele Napolitano, Mattia Bozzetti, Alessio Lo Cascio, Giuseppe De Stefano, Nicoletta Orgiana, Loris Riccardo Lopetuso, Antonio Maria D’Onofrio, Giovanni Camardese, Alfredo Papa, Franco Scaldaferri, Antonello Cocchieri, Davide Bartoli

**Affiliations:** 1 Department of Biomedicine and Prevention, Tor Vergata University, 00133 Rome, Italy; 2 CEMAD IBD Unit, Fondazione Policlinico Gemelli IRCCS, 00168 Rome, Italy; lorisriccardo.lopetuso@guest.policlinicogemelli.it (L.R.L.); alfredo.papa@policlinicogemelli.it (A.P.); franco.scaldaferri@policlinicogemelli.it (F.S.); 3 Direction of Health Professions, ASST Cremona, 26100 Cremona, Italy; mattia.bozzetti@asst-cremona.it; 4 Direction of Health Professions, La Maddalena Cancer Center, 90146 Palermo, Italy; 5 Division of Cardiology, Azienda Ospedaliero-Universitaria di Parma, University of Parma, 43126 Parma, Italy; cure.destefano@gmail.com; 6 CUT, Fondazione Policlinico Gemelli IRCCS, 00168 Rome, Italy; nicoletta.orgiana@policlinicogemelli.it; 7 Department of Life Science, Health, and Health Professions, Link Campus University, 00165 Rome, Italy; giovanni.camardese@policlinicogemelli.it; 8 Dipartimento Universitario di Medicina e Chirurgia Traslazionale, Università Cattolica del Sacro Cuore, 00168 Rome, Italy; 9 Department of Neuroscience, Section of Psychiatry, Fondazione Policlinico Universitario Agostino Gemelli IRCCS, 00168 Rome, Italy; antoniomdonofrio@gmail.com; 10 Section of Hygiene, Department of Health Science and Public Health, Università Cattolica del Sacro Cuore, 00168 Rome, Italy; antonello.cocchieri@policlinicogemelli.it; 11 Dipartimento di Medicina Molecolare e Clinica, Università Sapienza di Roma; 00185 Rome, Italy; bartoli.davide90@gmail.com

**Keywords:** inflammatory bowel disease, ulcerative colitis, Crohn’s disease, resilience, self-care

## Abstract

**Background/Objectives:** Inflammatory bowel disease (IBD), including Crohn’s disease (CD) and ulcerative colitis (UC), significantly affects patients’ quality of life. Resilience and self-care are vital for disease management, yet their relationship with IBD remains underexplored. This study investigates how self-care behaviours influence resilience in patients with IBD, taking into account sociodemographic and clinical factors. **Methods**: This was a multicentre observational study. Data were collected during routine outpatient visits between April and June 2024. Participants (≥18 years) with an IBD diagnosis for at least 12 months were recruited. Data were collected using validated instruments, including the Connor-Davidson Resilience Scale (CD-RISC-25), the Self-Care of Chronic Illness Inventory (SC-CII), and sociodemographic and clinical questionnaires. Disease activity was assessed using the Mayo Score (UC) and the Harvey–Bradshaw Index (CD). Descriptive, correlational, and regression analyses explored variable relationships. This study was conducted as part of the N-ECCO Research Grant initiative. **Results**: A total of 401 participants (CD: 196, UC: 205) were enrolled, with equal gender distribution (50.1% male). The descriptive analysis of self-care levels showed a mean score of 72.6 (*SD* = 12.5) for self-care maintenance, 81.0 (*SD* = 18.2) for self-care monitoring, and 70.5 (*SD* = 18.4) for self-care management. The UC patients had higher self-care management scores than the CD patients (*p* = 0.002). The median resilience score was 45, and self-care management positively predicted resilience (*β* = 0.041, *p* < 0.001). Disease severity negatively affected resilience and self-care, particularly in severe cases (*β* = −8.334, *p* < 0.001). The females reported higher resilience and self-care monitoring scores than the men. **Conclusions**: Resilience and self-care are interrelated and crucial in IBD management. Enhancing resilience through personalised nursing interventions and integrating psychological and educational support may improve self-care and clinical outcomes.

## 1. Introduction

The incidence of inflammatory bowel diseases (IBD), including Crohn’s disease (CD) and ulcerative colitis (UC), is increasing, particularly in recently industrialised countries. Global prevalence is expected to rise from 0.5% in 2010 to approximately 1% by 2030 [1]. This trend suggests that over ten million individuals in Western populations could be affected [2]. Despite therapeutic advances, IBD remains a chronic, incurable condition, with therapeutic objectives focusing on inducing and maintaining clinical remission, preventing disease-related complications, and enhancing patients’ quality of life [3,4]. Symptoms such as pain, fatigue, and gastrointestinal disturbances significantly impair quality of life and are major concerns for individuals with IBD [5,6]. Consequently, critical research priorities are improving quality of life and reducing mortality through comprehensive healthcare and nursing strategies [7,8].

IBD typically manifests in young adulthood, a period characterised by significant psychosocial development and the establishment of independent living [9]. The unpredictable and chronic nature of IBD can lead to psychological distress, social stigma, impaired social functioning, and worsening disease burden [10]. Symptoms such as faecal incontinence and frequent bowel movements may compromise patients’ dignity, resulting in social isolation and decreased self-esteem [11].

The European Crohn’s and Colitis Organisation (ECCO) recommends integrating psychological assessments and therapeutic interventions into IBD management practices to address these multifaceted challenges [12]. A key component of IBD management is self-care, which involves recognising symptoms, managing them, and adopting coping strategies [13]. Self-care practices among patients with IBD include dietary modifications, adherence to pharmacological treatments, regular physical activity, and participation in psychotherapy. However, current engagement in self-care among patients with IBD remains suboptimal, partly due to limited knowledge about effective self-management strategies and the diverse manifestations of the disease [14]. Research indicates that self-care involves disease management and the promotion of overall well-being and health, as articulated in the Middle Range Theory, which identifies self-care maintenance, self-care monitoring, and self-care management as its primary components [15].

Resilience, the ability to adapt positively to adversity, is crucial in managing chronic diseases such as IBD [16]. Higher resilience is associated with better stress management, treatment adherence, and overall quality of life [17]. Studies on chronic conditions such as diabetes, hypertension, kidney disease, heart failure, asthma, osteoarthritis, epilepsy, multiple sclerosis, HIV, and colon cancer suggest that greater resilience correlates with improved self-care, including symptom monitoring, treatment adherence, physical activity, and stress management. Interventional studies indicate that resilience-focused programmes may enhance self-care practices, though findings are mixed [18].

Szanton et al.’s theoretical framework integrates social, community, familial, individual, and physiological factors to explain resilience mechanisms. This model identifies resistance, recovery, and rebound as key stress management and self-care engagement elements [19]. Studies on specific conditions support these findings; for example, Schrijver et al. demonstrated that patients with chronic obstructive pulmonary disease with higher resilience exhibit greater self-care confidence and better quality of life. Additionally, Chang et al. found that resilience moderates the adverse effects of depressive symptoms on self-efficacy and self-care maintenance of heart failure patients [20,21].

Several studies have examined resilience’s impact on hospitalisation rates, opioid use, and psychological outcomes (anxiety and depression) in IBD populations [22,23,24]. Furthermore, resilience has been linked to positive thinking and effective disease self-management [17]. Self-efficacy and resilience are significant predictors for the successful transition of effective care from youth to adult in patients with IBD, consequently reducing disease stigma and increasing adherence to care [25]. Despite substantial evidence linking resilience and self-care in various chronic conditions, research examining this relationship in IBD remains limited. Understanding how resilience influences self-care behaviours in patients with IBD is essential for developing targeted interventions that improve disease management and patient outcomes [26].

## 2. Materials and Methods

### 2.1. Aim

This study explored the interaction between self-care and resilience in patients with IBD, identifying subgroup disparities to inform tailored, evidence-based nursing interventions that enhance self-care, resilience, and overall health outcomes.

### 2.2. Study Design and Setting

This research employed a multicentric cross-sectional design. The data were collected between April and June 2024 during routine outpatient visits. The participants were recruited from nine IBD outpatient clinics across Italy: four in the northern region, two in the central area, and three in the southern region. All centres were tertiary-level academic hospitals with dedicated IBD units staffed by multidisciplinary teams. This study was conducted in accordance with the Strengthening the Reporting of Observational Studies in Epidemiology (STROBE) guideline [27]. This publication is the result of the awarded N-ECCO Grant 2024.

### 2.3. Sample/Participants

A convenience sample of consecutive patients with IBD was recruited. The inclusion criteria required participants to be 18 years or older, have a confirmed IBD diagnosis, and provide informed consent. The exclusion criteria included an IBD diagnosis of less than 12 months, recent surgery (within 6 months), or the presence of another severe chronic condition.

### 2.4. Data Collection

Patients were consecutively enrolled after providing written informed consent. In cases where participation was declined, the next eligible patient was invited. The data were collected using self-administered questionnaires or, when necessary, through face-to-face interviews conducted by trained personnel. For this cross-sectional analysis, a minimum sample size of 200 participants was deemed sufficient to ensure adequate statistical power for multivariable regression analyses, following established recommendations for structural equation modelling, which suggest a minimum case-to-parameter ratio of 10:1 [26,28]. All individuals who met the inclusion criteria and consented to participate within this timeframe were included in the analysis. This larger-than-anticipated sample enhances the precision and generalisability of the study findings.

### 2.5. Sociodemographic and Clinical Questionnaire

The research team developed a comprehensive questionnaire to collect data, including sociodemographic information, age, gender, education, occupation, and geographical origin. The clinical variables encompassed previous hospitalisations, disease-related surgeries, past emergency department visits, disease duration, and the type of care provided at the enrolment centre.

### 2.6. The Self-Care Inventory

The Self-Care of Chronic Illness Inventory (SC-CII) is a comprehensive tool used to evaluate self-care across various chronic conditions [29,30]. It comprises three scales: self-care maintenance (7 items), self-care monitoring (5 items), and self-care management (6 items), as specified by Riegel et al. (2018) [30]. Each item is rated using a 5-point Likert scale, ranging from 0 (“never”) to 5 (“always”). The scores for the three domains are standardised on a scale of 0 to 100, with higher scores reflecting better self-care. A score of 70 or above indicates “adequate” self-care. The SC-CII is a valid and reliable instrument for assessing self-care in IBD [31].

### 2.7. The Self-Care Self-Efficacy Scale

The Self-Care Self-Efficacy Scale (SC-SES) measures patients’ confidence in managing self-care through 10 items scored on a 5-point Likert scale, with higher scores indicating greater self-efficacy [32]. Cross-cultural validation, including in Italy, has confirmed its strong reliability (Cronbach’s *α* = 0.92) and positive and construct validity. Higher SC-SES scores correlate with better SC-CII scores, confirming its relevance [29,30].

### 2.8. Resilience Index

The Connor–Davidson Resilience Scale (CD-RISC-25) was adopted to measure levels of resilience [33,34]. Its use has expanded to the general population for evaluating resilience and tracking changes post treatment. The CD-RISC 25 comprises 25 items across 5 factors: spiritual influences (items 3, 9, and 20), personal competence (items 14–19), intuitive confidence (items 11 and 21–25), life control (items 2 and 6–10), and acceptance of change (items 1, 4–5, and 12–13). Each item is scored on a 5-point Likert scale (0 = “not true at all” to 4 = “true nearly all the time”), with total scores ranging from 0 to 100. Higher scores indicate greater resilience. The scale is a validated tool for resilience assessment over time. The scale developers authorised the use of the CD-RISC 25. In accordance with the copyright policy, the required academic licensing fee for research use was paid.

### 2.9. Clinical Index

Disease activity was assessed using the Mayo score for UC and the Harvey–Bradshaw Index for CD. The Mayo score ranges from 0 to 12 and is categorised as follows: 0–2 = remission; 3–5 = mild; 6–10 = moderate; ≥10 = severe. The HBI includes five parameters—patient well-being, abdominal pain, number of liquid stools per day, abdominal mass, and presence of complications—and is interpreted as: <5 = remission; 5–7 = mild; 8–16 = moderate; ≥16 = severe [35]. Notably, biochemical markers complemented clinical indices to enable a more comprehensive assessment of disease activity. Biochemical remission was defined as C-reactive protein (CRP) levels of ≤0.5 mg/dL and faecal calprotectin levels of ≤250 µg/g, with both markers collected during routine IBD outpatient visits. These biochemical parameters were evaluated in conjunction with the clinical scores to define disease activity, as outlined in the study protocol. Based on the integrated clinical and biochemical data, the patients with IBD were classified into four categories of disease activity: remission, mild, moderate, or severe.

### 2.10. Statistical Analysis

Descriptive statistics were used to summarise the sample’s sociodemographic, clinical, and psychosocial characteristics, stratified by disease group. The continuous variables were reported as means and standard deviations (SDs) for the normally distributed data and medians with interquartile ranges (IQRs) for the non-normally distributed data. The categorical variables were summarised using frequencies and percentages. Normality of distribution was assessed using the Shapiro–Wilk test and visual inspection of histograms and residuals. The non-normally distributed variables were subsequently analysed using nonparametric analyses. For comparisons between groups, independent samples *t*-tests were used for the normally distributed continuous variables, and the Mann–Whitney U test was applied for the non-normally distributed data. Chi-square (χ^2^) tests were employed for the categorical variables, with Fisher’s exact test used when the cell counts were below five. Statistical significance was set at *p* < 0.05.

Correlations between continuous variables were assessed using Spearman’s rank correlation coefficient for non-normally distributed data. Multivariate regression analyses were conducted to identify predictors of key outcomes.

Based on data distribution, one-way ANOVA or Kruskal–Wallis tests were performed to examine associations between categorical variables and continuous outcomes. When significant differences were identified, pairwise comparisons with Bonferroni correction were used for posthoc analyses. Statistical analyses were conducted using R 4.3.3 [36], and all tests were two-tailed.

## 3. Results

A total of 452 eligible patients with IBD were approached consecutively during routine outpatient visits. Of these, 401 participants completed all the questionnaires and were included in the study, resulting in a response rate of 88.7%. The final sample (*n* = 401) had an almost equal gender distribution, with 50.1% males and 49.9% females. Gender distribution differed slightly between the CD and UC groups; males represented 52.6% of the CD group but only 47.8% of the UC group. The mean age of the sample was 42.89 years, with similar (*p* = 0.113) averages for CD (42.56 years, *SD* =16.66) and UC (43.20 years; *SD* = 16.24). Table 1 outlines the sociodemographic and clinical features of the sample.

### 3.1. Self-Care and Self-Efficacy Behaviours

The descriptive analysis of self-care levels showed that the mean score for self-care maintenance was 72.6 (*SD* = 12.5), while the mean score for self-care monitoring was 81.0 (*SD* = 18.2). Finally, the mean score for self-care management was 70.5 (*SD* = 18.4). The analysis of self-care and resilience provided a detailed understanding of how individuals with CD and UC manage their conditions. Variations in performance were observed across the three dimensions of self-care: maintenance, monitoring, and management. In self-care maintenance, no significant differences emerged between the groups. However, the CD patients exhibited slightly higher levels of engagement in routine health-sustaining behaviours than the UC patients (*U* = 18,215.5, *p* = 0.064).

Self-care monitoring revealed no statistically significant differences between the groups (*U* = 18,514.0, *p* = 0.103). The CD and UC patients demonstrated comparable capabilities, indicating robust monitoring practices throughout the population. However, notable differences were observed in self-care management. The UC patients outperformed CD patients in this aspect, suggesting stronger skills in effectively managing their condition (*U* = 16,479.0, *p* = 0.002).

Self-efficacy scores were similar across the total sample (median = 75.00, IQR = 20.00), with no significant differences between the patients with CD (75.00 [23.00]) and those with UC (75.00 [20.00]; *p* = 0.870). The median scores and comparisons are summarised in Table 2.

### 3.2. Resiliency Levels

Resiliency levels were analysed across the sample population. For comparative purposes, we categorised participants into “low resilience” (≤20th percentile of CD-RISC-25 score) and “high resilience” (≥80th percentile), based on distributional cut-offs validated in previous research on psychological outcomes in IBD and chronic illness populations [18,23]. This categorisation allows for the exploration of clinical and behavioural differences between psychologically vulnerable and resilient individuals.

Most participants (63.84%) demonstrated resilience levels ranging from sufficient to excellent. Nevertheless, nearly 20% of participants lacked resilience. The overall resilience score for the sample was *M* = 45 [21]. Among the dimensions of resilience, “Confidence in one’s intuition” achieved a higher score of *M* = 14 [6], while “Spiritual influences” recorded the lowest score, with *M* = 5 [3].

### 3.3. Correlations

Self-care maintenance showed significant positive associations with several variables. It was strongly correlated with self-care monitoring (*r* = 0.37, *p* < 0.001) and self-care management (*r* = 0.34, *p* < 0.001), indicating that individuals who engage in higher self-care and maintenance behaviours are more likely to excel in monitoring their health status and managing their conditions effectively. Furthermore, a moderate positive correlation was noted between self-care maintenance and resiliency (*r* = 0.14, *p* < 0.01), suggesting that sustaining consistent self-care behaviours may contribute to building psychological resilience. Self-care monitoring was significantly related to self-care management (*r* = 0.43, *p* < 0.001), reflecting the connection between these two dimensions. Resiliency exhibited significant relationships with both self-care maintenance (*r* = 0.14, *p* < 0.01) and self-care management (*r* = 0.29, *p* < 0.001). These findings imply that psychological resilience is supported by consistent maintenance behaviours and practical management skills, underscoring the importance of these factors in promoting better coping and adaptation in patients (Table 3).

### 3.4. Associations with Categorical Variables

Multiple ANOVAs revealed several significant associations between variables and grouping factors. Age demonstrated significant differences in self-care maintenance based on educational level (*χ*^2^ = 8.024, *p* = 0.046) and employment status (*χ*^2^ = 10.650, *p* = 0.031). Resilience was significantly associated with disease activity (*χ*^2^ = 12.025, *p* = 0.007). Self-efficacy showed a significant relationship with geographical area (*χ*^2^ = 7.609, *p* = 0.022). Significant differences were also noted in self-care monitoring and resilience, with women scoring higher in both areas. For self-care monitoring, the analysis yielded *W* = 17,746.5 (*p* = 0.041), and for resilience, *W* = 17,749.5 (*p* = 0.041).

### 3.5. Determinants of Resilience

The regression analyses investigated the predictors of resilience and its subdomains (spiritual influences, personal competence, confidence in one’s intuition, life control, and acceptance of change). The independent variables included in the models were determined using the AIC criterion. They consisted of self-care behaviours (self-care maintenance, self-care monitoring, and self-care management), educational level, disease activity, sex, years since diagnosis, previous hospitalisations, and prior surgeries. The first model, which evaluated spiritual influences, indicated that self-care management was a significant positive predictor (*β* = 0.019, *p* = 0.002), whereas other variables did not demonstrate substantial contributions. Self-care management (*β* = 0.026, *p* = 0.014) and self-care maintenance (*β* = 0.024, *p* = 0.050) were positive predictors of personal competence. Furthermore, disease activity adversely affected personal competence, with mild (*β* = −0.896, *p* = 0.046) and severe disease states (*β* = −1.640, *p* = 0.024) showing significant associations. Confidence in one’s intuition was notably influenced by self-care management (*β* = 0.041, *p* = 0.007) and was negatively associated with disease activity. Mild disease activity correlated with decreased confidence (*β* = −1.324, *p* = 0.033), while severe activity had a more pronounced negative effect (*β* = −2.343, *p* = 0.002). The model assessing life control indicated that self-care management (*β* = 0.041, *p* < 0.001) was a consistent positive predictor, whereas disease activity, particularly in severe cases (β = −2.785, *p* < 0.001), exerted a strong negative impact. Lastly, acceptance of change was similarly affected by self-care management (*β* = 0.041, *p* < 0.001) and negatively influenced by severe disease activity (*β* = −2.002, *p* = 0.002). All the models conducted are summarised in Table 4.

## 4. Discussion

This study highlights several significant findings regarding self-care and resilience among patients with UC and CD. A strong relationship was observed between resilience and self-care in patients with IBD. Among the self-care domains, self-care management showed the strongest correlation with resilience, underscoring its critical role in enabling patients to manage their condition actively. Patients with higher resilience developed more effective coping strategies, linking positive psychological adaptation to better clinical outcomes.

In our study, nearly a fifth of participants exhibited low resilience levels, underscoring the variability in patients’ capacity to cope with the challenges posed by their illness. Although we did not compare our sample with a control group, an important insight comes from a systematic review which found that patients with chronic physical diseases tend to have lower resilience scores than healthy individuals [37]. These results emphasise the importance of integrating resilience-building strategies into managing IBD, focusing on enhancing life control, self-efficacy, and adaptation, thereby helping to optimise clinical outcomes and improve patient’s quality of life. Similarly, an Italian study by Cococcia et al. (2021) reported a comparable resilience level in a monocentric outpatient sample. Although that study also focused on perceived stigma, which we did not assess, the consistency in resilience scores reinforces the generalisability of our findings to the Italian IBD population [38,39,40].

Furthermore, disease severity emerged as a significant negative predictor of resilience. This highlights how severe clinical manifestations may impair psychological resilience, limiting the ability to develop proactive coping strategies [41,42]. Symptoms, frequent hospitalisation, and an increased sense of loss of control negatively affect self-efficacy and resilience. Thus, targeted support combining psychological interventions with educational strategies may be required for patients with severe CD to enhance their resilience and self-care skills [43].

Our findings align with the existing literature. A systematic review by Jin et al. confirmed that resilience is positively associated with self-care in individuals with chronic conditions, facilitating better physical and emotional well-being [18]. Similarly, a scoping review by Chng et al. demonstrated that higher resilience correlates with reduced chronic pain, improved functional outcomes, better quality of life, and a lower risk of comorbid mental health disorders in chronic pain populations [44]. Further systematic reviews indicate that resilience is inversely related to depression, anxiety, incapacity, and somatisation, reinforcing its protective role against chronic disease progression [45]. Collectively, these findings suggest that while disease progression may not always be modifiable, resilience-enhancing interventions should be prioritised as a fundamental aspect of chronic disease management [46,47,48,49,50]. Moreover, resilience and self-care management are dimensions that operate in parallel. This mutual reinforcement indicates that improving one of these dimensions may also benefit the other, creating a positive cycle that supports physical and psychological well-being [51].

Sociodemographic differences significantly influence resilience and self-care practices. Women scored higher than men in resilience and self-care monitoring, suggesting greater awareness and proactive illness management [18,52]. In contrast, men may require specific interventions to boost their awareness and involvement in symptom monitoring. However, the literature on gender differences in resilience is not always consistent. For instance, a study by Bonanno and colleagues discovered that male participants were more likely to exhibit resilience compared to female participants, with female participants being less than half as likely to demonstrate resilience following trauma [53]. Similarly, other research has underscored that gender-related personality traits and coping flexibility play a moderating role in the effects of life event stress on psychosocial adjustment, with masculinity acting as a buffer in the relationship between stress and interpersonal functioning [54]. Likewise, in older populations, studies have indicated that older women are less likely to be resilient than older men [55]. Conversely, a comprehensive meta-analysis found no significant gender-based resilience differences, suggesting gender may not universally determine resilience levels [56]. These findings indicate that tailored healthcare interventions should account for gender-related differences in resilience and self-care engagement [57,58].

**Figure 1:**
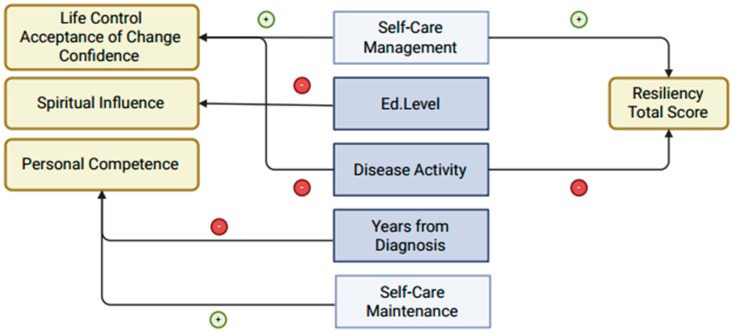
Conceptual representation of significant predictors of resilience in patients with IBD. Arrows indicate statistically significant associations (*p* < 0.05) between independent variables (**centre**), domains of resilience (**left**), and overall resilience (**right**). Green arrows (+) represent positive associations, while red arrows (–) indicate negative associations. Self-care management is positively associated with multiple domains of resilience, whereas higher levels of disease activity show negative associations across several aspects of resilience.

Interrelationships among self-care dimensions indicate that self-care maintenance, monitoring, and management positively correlate with resilience (Figure 1). Individuals proficient in one aspect of self-care often excel in others, highlighting their interdependent nature. For instance, a study on heart failure patients found that self-care maintenance and management are closely linked, supporting the idea that these dimensions function synergistically [59]. Effective self-care in chronic conditions relies on integrating maintenance, monitoring, and management. Self-care maintenance involves consistent health-promoting behaviours, self-care monitoring entails tracking health status changes, and self-care management requires active decision-making and problem-solving [60]. Together, these processes improve health outcomes and quality of life. From an advanced practice nursing perspective, these findings reinforce the importance of a holistic, multidisciplinary approach to patient care. Nurses in IBD management should collaborate with psychologists, dietitians, and gastroenterologists to develop comprehensive patient-centred interventions. Implementing structured self-care and resilience training programmes within clinical practice could help bridge the physical and psychological well-being gap, fostering a more sustainable approach to chronic disease management [61,62,63,64,65]. Nurse-led interventions, including structured education programmes, motivational interviewing, and cognitive behavioural strategies, can enhance patients’ self-efficacy and resilience, ultimately improving their disease management capabilities. IBD nurses, in particular, play a crucial role in patient education, medication adherence, and psychosocial support, all of which contribute to building resilience and optimising self-care strategies [37,39,40].

Higher resilience levels correlated with improved self-care across all dimensions. Studies support this relationship: resilience is positively linked to self-care in chronic conditions, diabetes, and older adults with multiple conditions [18]; higher resilience is associated with better self-care activities in people with diabetes [66]; resilience is positively related to self-care dimensions in older adults with multiple chronic conditions [18]; and self-care, adherence to treatment, and other factors directly related to physical illness are associated with resilience in physically ill people [67]. It is worth noting, however, that not all findings are consistent. One study found no significant associations between resilience and self-care in heart failure patients, suggesting that context and measurement tools may influence outcomes [68]. Overall, these findings underscore the necessity of integrating self-care and resilience-building interventions into clinical practice [69]. By implementing educational workshops, peer support programmes, and resilience coaching, nurses can empower patients with IBD to develop stronger coping mechanisms, leading to improved self-care and long-term disease management [17].

Self-management abilities varied across educational and occupational contexts. Additionally, geographical differences in self-efficacy emerged, suggesting regional disparities in perceived competence. These findings align with the broader literature, which consistently identifies sociodemographic factors as crucial determinants of self-care practices. For instance, higher socioeconomic status is linked to better self-care in heart failure patients [70]. In contrast, marital status, employment, and lifestyle factors have been linked to self-care behaviours [71,72]. Similarly, age, income, and education are frequently cited as determinants of health status and self-care practices [73,74,75]. Interestingly, Sedlar and colleagues also emphasise the influence of depression, underscoring the complex interplay between mental health and self-care management.

In contrast, some studies suggest that these sociodemographic factors have a minimal influence on self-care behaviours. For example, Fredericks and colleagues argue that sociodemographic characteristics and health profiles exert limited impact on self-care performance [76,77]. However, this divergence may be explained by variations in study populations, methodologies, or the specific conditions examined, suggesting that while sociodemographic factors are often influential, their role may be modulated by additional variables such as disease type, mental health status, and regional or cultural differences.

This study presents several important limitations that must be acknowledged. A key limitation lies in the absence of a control group, which significantly constrains the ability to contextualise the resilience levels and self-care behaviours observed in patients with IBD. Without comparisons to healthy individuals or patients with other chronic conditions, it remains unclear whether the identified resilience scores reflect disease-specific challenges or more generalised patterns seen in chronic illness. Future studies should explicitly include such control groups to allow for more meaningful interpretation of findings and to better understand the unique psychosocial profiles of the IBD population.

Additionally, the cross-sectional design of the present study imposes a major constraint on the interpretation of the associations between resilience and self-care. While our findings support the existence of a statistically significant relationship, the design precludes any inference of causality. Therefore, longitudinal studies are strongly recommended to validate the directionality and stability of these associations over time and in varying clinical contexts. Such designs would also be instrumental in clarifying whether resilience functions as a precursor, consequence, or co-occurring factor in relation to self-care behaviours in chronic disease management.

Although our study did not involve any interventional components, the observed associations suggest that targeted interventions aimed at enhancing resilience may offer clinical benefits, particularly for patients demonstrating lower resilience scores. Future randomised controlled trials should investigate the effectiveness of evidence-based approaches such as cognitive behavioural therapy (CBT), mindfulness-based stress reduction (MBSR), and structured self-care education programmes. These interventions have already shown promising outcomes in other chronic disease populations and could be adapted to the specific needs of patients with IBD to promote better psychosocial functioning and self-management capacities [21,39,40]. Despite these limitations, this study offers valuable insights with clinical implications. This study presents some limitations. First, it includes only cross-sectional patient-level data. Given the cross-sectional nature of this study, causality cannot be established, and the relationship between these constructs may be bidirectional.

The identified relationship between resilience and self-care underscores the need for tailored interventions to enhance psychological resilience and address specific self-care challenges in patients with IBD. Nurses in IBD management should collaborate with psychologists, dietitians, and gastroenterologists to develop comprehensive patient-centred interventions. Implementing structured self-care and resilience training programmes within clinical practice could help bridge the physical and psychological well-being gap, fostering a more sustainable approach to chronic disease management [61]. Future research should focus on developing resilience-based interventions and assessing their impact on self-care, disease management, and quality of life. Such initiatives could bridge the gap between research and clinical practice, fostering improved holistic care.

The findings underscore the critical need to incorporate patient education systematically into routine clinical practice. Tailored educational programmes have the potential to improve disease comprehension, foster self-care behaviours, and empower individuals to manage their chronic condition more effectively. In parallel, interventions that build resilience play a key role in helping patients cope with physical and emotional challenges, contributing to improved quality of life. These insights highlight valuable areas for future nursing interventions to enhance resilience and self-care among patients with IBD. Nevertheless, these recommendations should be interpreted cautiously, as they are not based on intervention data. Future research—including caregiver perspectives and longitudinal follow-up—will be essential to inform more robust and evidence-based clinical strategies.

## 5. Conclusions

This study underscores the critical role of resilience and self-care in managing IBD. Higher resilience correlates with better self-care maintenance, monitoring, and management. However, nearly a fifth of participants exhibited low resilience, necessitating targeted interventions. Variations in self-care and resilience by gender, disease type, and geographic region highlight the importance of tailored, evidence-based healthcare workers’ strategies. Enhancing resilience through targeted interventions, supported by healthcare workers and other specialists, could improve coping, treatment adherence, and overall well-being, leading to better outcomes in IBD management.

## Figures and Tables

**Table 1:** Sociodemographic characteristics and clinical features.

Variables	Total (*n* = 401)	CD (*n* = 196)	UC (*n* = 205)
Gender *n* (%)			
Male	201 (50.12%)	103 (52.55%)	98 (47.80%)
Female	200 (49.88%)	93 (47.45%)	107 (52.20%)
Age (y) M (*SD*)	42.89 (16.43)	42.56 (16.66)	43.20 (16.24)
Educational level *n* (%)			
First school	13 (3.24%)	5 (2.55%)	8 (3.90%)
Middle school	74 (18.45%)	30 (15.30%)	44 (21.46%)
High school diploma	183 (45.64%)	101 (51.53%)	82 (40.00%)
Bachelor’s degree	131 (32.67%)	60 (30.61%)	71 (34.63%)
Occupation *n* (%)			
Retired	44 (10.97%)	22 (11.23%)	22 (10.73%)
Homemaker	25 (6.23%)	13 (6.64%)	12 (5.86%)
Student	53 (13.21%)	27 (13.78%)	26 (12.69%)
Employed	251 (62.59%)	120 (61.23%)	131 (63.91%)
Unemployed	28 (6.98%)	14 (7.15%)	14 (6.83%)
Geographical area *n* (%)			
North	135 (33.67%)	67 (34.19%)	68 (33.18%)
Centre	157 (39.16%)	92 (46.94%)	65 (31.71%)
South and islands	109 (27.19%)	37 (18.88%)	72 (35.80%)
Disease activity (*n*%)			
Remission	179 (44.6%)	102 (52%)	77 (37.6%)
Mild	146 (36.4%)	68 (34.7%)	78 (38%)
Moderate	63 (15.7%)	24 (12.2%)	39 (19%)
Severe	13 (3.2%)	2 (1%)	11 (5.4%)
Previous hospitalisations for disease-related causes *n* (%)			
No	146 (36.41%)	64 (32.66%)	82 (40.00%)
One	106 (26.44%)	47 (23.98%)	59 (28.79%)
More than one	149 (37.16%)	85 (43.37%)	64 (31.22%)
Surgeries for disease-related causes *n* (%)			
No	279 (69.58%)	99 (50.52%)	180 (87.81%)
One	60 (14.97%)	50 (25.52%)	10 (4.88%)
More than one	62 (15.47%)	47 (23.98%)	15 (7.32%)
Emergency department visits in the last 12 months *n* (%)			
No	272 (67.84%)	145 (73.98%)	127 (61.96%)
1–2 times per year	105 (26.19%)	42 (21.43%)	63 (30.74%)
3–4 times per year	20 (4.99%)	7 (3.58%)	13 (6.35%)
5 or more times per year	4 (1.00%)	2 (1.03%)	2 (0.98%)
Time from diagnosis (y) M (SD)	11.39 (9.35)	11.22 (9.96)	11.55 (8.74)
Care at the enrolment centre (y) M (SD)	5.82 (5.57)	6.21 (5.94)	5.45 (5.18)

CD, Crohn’s disease; UC, ulcerative colitis; M, median; SD, standard deviation. Disease activity was classified into remission, mild, moderate, or severe based on the Mayo score (UC), the Harvey–Bradshaw Index (CD), and biochemical markers (CRP ≤ 0.5 mg/dL, faecal calprotectin ≤ 250 µg/g).

**Table 2:** Descriptive statistics of CD and UC patients across scores.

	Total Sample	CD	UC	
Variables	Median [IQR]	Median [IQR]	Median [IQR]	Sign.
Resiliency	45.00 [21.00]	44.00 [19.00]	45.00 [18.00]	0.205
Spiritual influences	5 [3]	5 [3]	5 [3]	0.521
Personal competence	9 [5]	9 [4]	9 [5]	0.645
Confidence in one’s intuition	14 [6]	13 [6]	14 [6]	0.060
Life control	8 [7]	7 [6.25]	8 [6.00]	0.277
Acceptance of change	9 [5]	9 [6]	9 [6]	0.235
Self-efficacy	75.00 [20.00]	75.00 [23.00]	75.00 [20.00]	0.870

IQR, interquartile range, CD, Crohn’s disease; UC, ulcerative colitis.

**Table 3:** Correlation matrix.

	Age	SC—Maintenance	SC—Monitoring	SC—Management	Self-Efficacy	Spiritual Influences	Personal Competence	Confidence in One’s Intuition	Life Control	Acceptance of Change
Age										
SC–Maintenance	−0.10									
SC–Monitoring	0.01	0.33 ***								
SC–Management	0.02	0.32 ***	0.41 ***							
Self-Efficacy	−0.01	−0.03	−0.01	−0.07						
Spiritual influences	0	0.09	0.05	0.2 ***	−0.01					
Personal competence	0.03	0.15 **	0.06	0.2 ***	−0.03	0.67 ***				
Confidence in one’s intuition	0.03	0.14 **	0.11 *	0.22 ***	0	0.66 ***	0.83 ***			
Life control	0.06	0.13 **	0.11 *	0.27 ***	−0.03	0.67 ***	0.54 ***	0.59 ***		
Acceptance of change	0.01	0.11 *	0.08	0.24 ***	0.02	0.65 ***	0.58 ***	0.62 ***	0.76 ***	

Significant at * *p* < 0.05, ** *p* < 0.01, and *** *p* < 0.001.

**Table 4:** Regression analyses.

	Spiritual Influence		Personal Competence		Confidence in One’s Intuition		Life Control		Acceptance of Change		Resiliency	
Variable	*β*	*p* Value	*β*	*p* Value	*β*	*p* Value	*β*	*p* Value	*β*	*p* Value	*β*	*p* Value
(Intercept)	5.105	0.000	7.607	0.000	11,100	0.000	6.690	0.000	7.579	0.000	38,932	0.0000 ***
Self-Care Management	0.019	0.002	0.026	0.014 **	0.041 *	0.007 **	0.041	0.000 ***	0.041	0.000 ****	0.132	0.001 **
Self-Care Maintenance	0.006	0.369	0.024	0.050 *	0.029	0.091 **	0.021	0.103	0.014	0.280	0.084	0.067
Educational Level (Middle school)	−0.613	0.370	0.134	0.917	−0.120	0.942	−0.623	0.572	−0.685	0.595	−2.151	0.593
Educational Level (High school diploma)	−1.266	0.043 *	−0.545	0.651	−1.323	0.387	−1.656	0.100	−1.388	0.249	−5.401	0.134
Educational Level (Bachelor’s Degree)	−0.912	0.157	−0.468	0.707	−0.649	0.679	−0.897	0.390	−0.904	0.465	−4.051	0.273
Disease activity (Mild)	−0.485	0.064	−0.896	0.046 *	−1.324	0.033 *	−1.209	0.013 *	−1.120	0.016 *	−4.479	0.010 **
Disease activity (Moderate)	−0.768	0.027 *	−0.902	0.109	−1.258	0.109	−1.825	0.002 **	−1.557	0.009 **	−5.457	0.010 **
Disease activity (Severe)	−1.112	0.001 **	−1.640	0.024 *	−2.343	0.002 **	−2.785	0.000 ***	−2.002	0.002 **	−8.334	0.000 ***
Sex (0 = female; 1 = male)	0.211	0.366	0.511	0.202	0.976	0.083	0.324	0.434	0.451	0.276	2.851	0.068
Years from diagnosis	0.004	0.770	0.050 *	0.021 *	0.035	0.242	0.017	0.586	0.016	0.520	0.094	0.292
Hospital admission (0 = no; 1 = yes)	0.046	0.768	−0.409	0.127	−0.379	0.294	−0.078	0.787	−0.114	0.662	−1.052	0.279
Previous surgeries (0 = no; 1 = yes)	−0.217	0.201	−0.358	0.211	−0.693	0.057	−0.336	0.242	−0.276	0.299	−1.383	0.142
Model												
Test F	F_(12)_ = 3.213, *p* < 0.0001		F_(12)_ = 3.798, *p* < 0.0001		F_(12)_ = 4.180, *p* < 0.0001		F_(12)_ = 3.930, *p* < 0.0001		F_(12)_ = 3.930, *p* < 0.0001		F_(12)_ = 3.914, *p* < 0.0001	

Significant at * *p* < 0.05, ** *p* < 0.01, and *** *p* < 0.001.

## Data Availability

The datasets used and/or analysed during the current study are available from the corresponding author on reasonable request.

## Abbreviations

The following abbreviations are used in this manuscript:

IBD	inflammatory bowel disease
CD	Crohn’s disease
UC	ulcerative colitis
ECCO	European Crohn’s and Colitis Organisation
STROBE	Strengthening the Reporting of Observational Studies in Epidemiology
SC-CII	Self-Care of Chronic Illness Inventory
SC-SES	Self-Care Self-Efficacy Scale
CD-RISC-25	Connor-Davidson Resilience Scale
SD	standard deviation
